# Regulatory CD4^+^CD25^+^ T Cells Dampen Inflammatory Disease in Murine Mycoplasma Pneumonia and Promote IL-17 and IFN-γ Responses

**DOI:** 10.1371/journal.pone.0155648

**Published:** 2016-05-13

**Authors:** Adam N. Odeh, Jerry W. Simecka

**Affiliations:** Preclinical Services and the Department of Pharmaceutical Sciences, UNT System College of Pharmacy, University of North Texas Health Science Center, Fort Worth, TX, 76107, United States of America; The University of Melbourne, AUSTRALIA

## Abstract

Mycoplasmas cause respiratory diseases characterized by persistent infection and chronic airway inflammation. Mycoplasma lung disease is immunopathologic, with CD4^+^ Th cells determining both disease severity and resistance to infection. Th2 cell responses promote immunopathology, while Th1 cells confer resistance to infection. However, regulatory CD4^+^ T cells may also have a role in the pathogenesis of mycoplasma respiratory diseases. We hypothesized Treg cells control the severity of the inflammatory lesions and may also promote persistence of infection. To examine this, BALB/c mice were depleted of CD25^+^ cells, and had increased disease severity due to *Mycoplasma pulmonis* infection. Increases in mycoplasma antibody responses and lymphocyte infiltration into lungs also occurred after CD25^+^ cell depletion. CD4^+^CD25^+^ regulatory T cells promoted IFN-γ and IL-17 mycoplasma-specific CD4^+^ T cell responses *in vitro* and *in vivo*, while dampening IL-13^+^ Th responses. Neither IL-10 nor TGF-ß expression was detected in CD4^+^CD25^+^ T cells from lymph nodes. Thus, a regulatory T cell population plays an important role in controlling damaging immune responses in mycoplasma respiratory disease but does not contribute to persistence of infection. It appears that a regulatory T cell population preferentially dampens Th2 cell-mediated inflammatory responses to mycoplasma through a mechanism independent of IL-10 or TGF-ß characteristic of “classic” Treg cells.

## Introduction

Mycoplasmas cause respiratory diseases in humans and animals. In humans, *Mycoplasma pneumoniae* causes up to 30% of all community-acquired pneumonia cases each year, and is commonly referred to as “walking pneumonia” [1]. Mycoplasma respiratory diseases are typically characterized by high morbidity and low mortality, with infections persisting for weeks, some requiring hospitalization (more than 100,000 people each year). Mycoplasma infections are also linked with exacerbation of a number of other diseases, including increased severity of asthma and certain autoimmune conditions [2–4]. In general, mycoplasma infections are persistent and lead to the development of the chronic inflammatory lesions along the airways. Previous work using the murine pathogen *Mycoplasma pulmonis* has revealed that a large component of the immune response is immunopathologic [5, 6], and T cell responses and their regulation are critical in determining the severity of disease [7–9]. In particular, Th2 cell responses contribute to increased disease severity [10]. Although other cell populations can modulate mycoplasma disease [7, 11, 12], the role of Treg cells in mycoplasma respiratory diseases has not yet been examined.

Regulatory T cells are composed of several subpopulations of T cells, including specialized subsets of CD4^+^ T cells, whose major functions include the suppression or dampening of immune responses [13]. These cell populations are able to limit the severity of inflammatory responses and prevent the development of immunopathology. Although the variety of suppressive mechanisms used by regulatory T cells are still being defined, cytokine secretion appears to be one of the main methods of control. T regulatory (Treg) cells are one of the most studied of these cell populations and are typically identified as CD4^+^CD25^+^FoxP3^+^ T cells. Several studies found that Treg cells produce both interleukin-10 (IL-10) and transforming growth factor-β (TGF-β), which is central to their ability to suppress cell proliferation and activation [14–22]. However, recent studies suggest that populations of Treg cells are capable of producing other cytokines, e.g. IL-17 and IFN-γ, which may also participate in the function of these cells [23–32]. These studies suggest that the conventional model, which holds that Treg cells dampen immune responses through secretion of IL-10 and/or TGF-β, may be oversimplified, and it also demonstrates that the mechanisms through which any regulatory T cell population can act may vary depending on the types of immune and inflammatory responses generated.

We are unaware of studies examining the role of Treg and related cells in mycoplasma diseases. There are some studies examining the role of Treg cell activity in pulmonary infections, and it is clear that modulation of Treg cell activity in some cases benefits the host and in other cases benefits the pathogen [33–35]. Consistent with work on the role of Treg cells in autoimmune diseases, Treg cells limit the damage to the eyes and the liver in murine models of herpes simplex ocular and *Schistosoma mansoni* chronic infections, respectively [36, 37]. In contrast, the activity of regulatory T cells may promote the development of chronic or persistent infections through immune system suppression. In this case, removal of Treg cells or blockade of their suppressive activity may ultimately lead to reduced disease severity and lower numbers of or clearance of the infectious agent. For example, this occurs in infections due to *Mycobacterium tuberculosis* [38], *Leishmania major* [39], *Plasmodium yoelii* [40, 41], *Plasmodium falciparum* [40, 41], and *Salmonella* [42]. Therefore, the impact of regulatory T cell populations on the progression of infectious diseases can vary and can be unpredictable. However, the role of regulatory T cell populations in infectious disease most likely depends on the mechanisms through which these cells act and impact the host responses to the infection.

Given the persistence of mycoplasma infections and the development of chronic inflammatory lesions, it was hypothesized that regulatory T cells control the severity of the inflammatory lesions through production of IL-10 or TGF-ß, but in doing so, the activity of these cells inadvertently promote persistence of infection, as found in other diseases. To address this hypothesis, we examined the role of regulatory T cells in murine mycoplasma pneumonia due to the natural pathogen, *M*. *pulmonis*. The studies presented here demonstrate that CD25^+^ regulatory T cells do play an important role in controlling damaging immune responses in mycoplasma respiratory infection but do not contribute to persistence of infection. In examining potential mechanisms, CD4^+^CD25^+^ T cells did not produce IL-10 or TGF-ß, suggesting an alternative mechanism for their activity. In support, CD4^+^CD25^+^ T cells from mycoplasma infected mice reduced Th2 cell responses and promoted the secretion of IFN-γ and/or IL-17 by other cell types *in vitro* and *in vivo*. These findings suggest that regulatory T cells dampen Th2 cell-mediated inflammatory disease associated with chronic pulmonary disease due to mycoplasma infection.

## Materials and Methods

### Mice

Female Balb/cAnNHsd wild-type mice, tested to be virus- and mycoplasma-free, were obtained from Harlan Laboratories (Indianapolis, IN). Mice were housed in sterile microisolator cages supplied with sterile bedding, with food and water provided ad libitum. Mice used in the study were between 6 and 8 weeks of age. Female mice were used in all studies. Prior to experimental infection, mice were anesthetized i.p. with diluted ketamine-xylazine. After infection, mice were monitored at least every two days for changes in health. Loss of body weight and clinical signs (e.g. ruffled fur) did occur. No measures were taken to alleviate any impact on health of infected mice. All animal studies were reviewed and approved by the University of North Texas Health Science Center Institutional Animal Care and Use Committee.

### Mycoplasma

The UAB CT strain of *M*. *pulmonis* was used in all experiments. Stock cultures were grown as previously described in mycoplasma pleuropneumonia-like organism medium (Acumedia, Lansing, MI) and frozen in 1-ml aliquots at -80°C [43]. For inoculation, thawed aliquots were dilute to 2–5 x 10^5^ CFU/20 μl. Intranasal inoculations of 20 μl of diluted mycoplasma were given under light anesthesia for experimental infections.

### Preparation of *M*. *pulmonis* Ag

Crude preparations of *M*. *pulmonis* membrane were used for *in vitro* stimulation of cells and prepared as previously described [44]. Briefly, *M*. *pulmonis* was cultured at 37°C in mycoplasma broth medium and harvested at pH 7. Cells were centrifuged at 10,000 rpm for 20 minutes, and resuspended in 5 ml sterile 0.25 M NaCl. Cells were centrifuged again, and resuspended in 4 ml of 2M glycerol at 37°C. Cells were then sonicated at the highest possible setting for 15 seconds using a Vibra cell sonicator (Sonics & Materials/Vibrio Cell, Newtown, CT), and incubated at 37°C for 10 minutes. Cells were then forced through a 27-gauge needle in 0.5 ml amounts into 25 ml aliquots of distilled water. Unlysed organisms were removed by a 20-minute centrifugation at 10,000 rpm. Supernatants containing the actual membrane Ag were centrifuged at 20,000 rpm for 1 hour. Membrane Ag was then resuspended in sterile PBS (HyClone Laboratories, Logan, UT) and stored at -80°C. Protein concentrations were determined using a Bradford protein assay (Bio-Rad, Hercules, CA).

### CD25^+^ T cell depletion

Similar to previous studies [21, 36, 39], mice were given intraperitoneal injections of anti-CD25 (PC61) antibody (BioXCell, West Lebanon, NH), 0.5 mg per mouse (100 μl), one day prior to infection, and again 6 days after infection. One dose of this antibody can lead to greater 80% reduction of CD25^+^ T cells in peripheral lymph nodes and spleens [39]. After 14 days of treatment, similar levels of depletion were confirmed by staining lymphocytes from lower respiratory lymph nodes (LRN) and lungs with PerCP-labeled anti-CD4 monoclonal antibody (BD Pharmingen, San Diego, CA), PE/Cy7-labeled anti-CD25 mAb (Abcam, Cambridge, MA), and APC-labeled anti-FoxP3 mAb (eBioscience, San Diego, CA) followed by flow cytometry. Control mice were injected with 100 μl sterile PBS or isotype control Ab.

### Determination of mycoplasma numbers

The numbers of CFU in the lungs were determined as previously described [45]. Briefly, lungs were placed in 1 ml of mycoplasma broth medium and minced. Samples were sonicated (Vibra cell sonicator; Sonics & Materials/Vibro Cell) for 1 minute at 50 amplitudes without pulsing. Serial dilutions were prepared, and 20 μl of each dilution was plated onto mycoplasma agar medium. Plates were incubated at 37°C for 7 days. Colonies were then counted and CFU were calculated.

### Assessment of gross lesions

Lungs were removed, and each lobe was examined individually for the presence of gross lesions. The percentage of gross lesions on each lobe was estimated and recorded. Overall gross lesion scores were calculated with each score weighted by the percentage that each lobe contributes to the total lung weight, as previously described [46].

### Histopathology

Lungs were fixed in alcohol formalin (4% glacial acetic acid (Fisher Scientific), 6% formaldehyde solution (Fisher Scientific), 40% deionized water, and 50% of 95% ethanol). Tissues were embedded in paraffin, sectioned at a thickness of 5 μm, and stained with H&E for light microscopy by HSRL, Inc. (Mount Jackson, VA). Each lung lobe was sectioned separately. Histology slides were scored for lesion severity (scale of 0–4) on the basis of the characteristic lesions of murine respiratory mycoplasmosis, as described previously [47]. Scores refer to: 1) peribronchial and perivascular lymphoid hyperplasia or infiltration (peribronchial infiltrate), or submucosal infiltrate in nasal passages; 2) neutrophilic exudate in airway lumina (airway exudate); 3) hyperplasia of airway mucosal epithelium (epithelial); 4) mixed neutrophilic and histiocytic exudate in alveoli (alveolar exudate). A score for each lesion was weighted according to the percentage each lobe contributes to the total lung weight in arriving at a total lesion score for each set of lungs. For each of the four lesions, a lesion index was calculated by dividing the observed lesion score by the maximum lesion score possible. Thus, the maximum lesion index possible for any lesion was 1.0. The overall lesion index was the mean of the four lesion indices in a lung.

### Lymphocyte Isolation

Mononuclear cells from lungs were isolated as previously described, with minor modifications [7, 48]. Briefly, each set of lung lobes was dissected and placed into GentleMACS C tubes (Miltenyi Biotec, Auburn, CA) containing RPMI 1640 medium (HyClone Laboratories), 300 U/ml *Clostridium histolyticum* type I collagenase (Worthington Biochemical, Freehold, NJ), 50 U/ml DNase (Sigma-Aldrich, St. Louis, MO), 5% FBS (Hyclone Laboratories), 10 mM HEPES (Fisher Scientific, Pittsburgh, PA), and antibiotic/antimycotic solution (Cellgro, Manassas, VA). Lung samples were homogenized using a GentleMACS (Miltenyi Biotec) on the provided setting for mouse lungs, protocol 2. Homogenates were then incubated at 37°C while mixing on a Nutator (Fisher Scientific) for 20 minutes. Subsequently, the homogenates were filtered through a 250-μm nylon mesh. Cells were then purified through density-gradient centrifugation using Lympholyte M (Cedarlane Laboratories, Burlington, NC).

Spleen and lower respiratory lymph nodes were pushed through a 250-μm nylon mesh, and cells isolated through centrifugation. This was followed by red cell lysis using ammonium chloride-potassium carbonate lysis buffer, or ammonium chloride-Tris lysis buffer. Total cells were counted using a Cellometer Auto T4 cell counter (Nexcelom Bioscience, Lawrence, MA).

### Flow cytometric analysis

Isolated cells were blocked with Fc block (anti-CD16/32; BD Pharmingen) and/or unconjugated Streptavidin (Invitrogen, Carlsbad, CA) and cell surface staining was performed on live cells resuspended in PBS containing 2% FBS. For intracellular FoxP3 staining, cells were fixed and permeabilized according to manufacturer’s instructions using fixation/permeabilization solution (eBioscience). For intracellular cytokine staining, cells resuspended in culture medium [RPMI 1640 medium (HyClone Laboratories) containing 10% FBS (HyClone Laboratories), 10 mM HEPES (Fisher Scientific), antibiotic/antimycotic (Cellgro), and 0.005% 2-mercaptoethanol (Sigma-Aldrich)]. Cells were treated with mycoplasma membrane Ag (5 μg/ml) overnight at 37°C with 5% CO_2_. The next day cells were treated with 50 ng/ml phorbol 12-myristate 13-acetate (PMA; Sigma-Aldrich), and 500 ng/ml ionomycin (EMD, Gibbstown, NJ). Cells were incubated for 1 hour, then treated with GolgiPlug, containing brefeldin A (1 μl/ml) (BD Pharmingen). Cells were incubated for 4 more hours.

The following antibodies were used for staining: anti-IFN-γ-Alexa Fluor (AF) 488, anti-IL-13-PE, anti-F4/80-PE, anti-FoxP3-allophycocyanin (APC), anti-IFN-γ-APC, anti-CD127-APC/AF750 (eBioscience), anti-CD11c-AF488, streptavidin-PE/AF610 (Invitrogen), anti-CTLA-4-PE, anti-CD4-peridinin chlorophyll-α protein (PerCP), anti-CD8-PerCP (BD Pharmingen), anti-CD62L-biotin, anti-CD25-PE/Cy7, anti-DX5-APC/Cy7 (Abcam), anti-CD3-PE/Cy7, anti-B220-APC, anti-GITR-AF700, anti-IL-17-AF700, and anti-CD44-APC/Cy7 (Biolegend, San Diego, CA). Cells were analyzed using a BD LSRII Flow Cytometer and BD FACSDiva Software (BD, Fullerton, CA). Further data analysis was performed using FlowJo flow cytometry analysis software (Tree Star, Ashland, OR). To determine the total number of cells in a specific population, the percentages of stained cells were multiplied by the total numbers of cells recovered.

### Cell separations

Cells were isolated using paramagnetic bead-conjugated antibodies and AutoMACS (Miltenyi Biotec) following manufacturer’s instructions. Treg cells and CD4^+^CD25^-^ Th cells were isolated in a two-step process. First, CD4^+^ cells were isolated by negative selection. Cells were treated with a cocktail of biotin-anti-CD8, biotin-anti-CD11b, biotin-anti-CD45R, biotin-anti-CD49b, and biotin-anti-Ter-119, followed by anti-biotin magnetic microbeads. These labeled cells were depleted from the samples, and the remaining cells were treated with anti-CD25-PE mAb. Cells were then treated with anti-PE magnetic microbeads and separated into two fractions: CD4^+^CD25^+^ Treg cells, and CD4^+^CD25^-^ Th cells. For *in vitro* cultures, whole splenocytes were treated with biotin-anti-CD3, followed by anti-biotin magnetic microbeads. CD3^+^ cells were depleted from the samples, and remaining cells were used as antigen-presenting cells (APC). This approach for cell isolation was found to result in greater than 95% purity using flow cytometry.

### *In vitro* cultures

96-well plates were incubated overnight at 37°C with 0.5 μg/ml anti-CD3 antibody (BD Pharmingen) in culture medium. Treg cells, CD4^+^ Th cells, or APCs were isolated as described in the previous section (Cell separations). 10^5^ of each kind of cell suspended in culture medium were seeded into the wells of a 96-well plate. Samples in anti-CD3 coated wells were treated with anti-CD28 antibody (2.5 μg/ml) (BD Pharmingen), and incubated at 37°C, 5% CO_2_ for 4 days. Other samples were treated with APCs and 5 μg/ml of mycoplasma membrane antigen. These plates were incubated for 6 days, and supernatants were collected.

### Cytokine measurements

Levels of IFN-γ, IL-4, IL-10, IL-13, and IL-17 were measured in the supernatants from the *in vitro* cultures. Cytokine levels were measured using a custom Bio-Plex kit (Bio-Rad), and 96-well filter-bottom plates. Samples were analyzed according to manufacturer’s instructions. Final readings were obtained using a Bio-Plex 100 system (Bio-Rad, Hercules, CA), and actual cytokine concentrations were determined by comparison with standard curves generated from murine recombinant cytokines. Data were analyzed using Bio-Plex Manager software (Bio-Rad).

The levels of TGF-β in supernatants were measured by capture ELISA, using an eBioscience Ready-SET-Go ELISA kit (eBioscience). Briefly, Probind 96-well flat-bottom microtiter plates (BD Biosciences) were coated overnight at 4°C with capture antibody. Plates were washed with PBS/0.01% Tween 20 and blocked with assay diluent. Supernatant samples were treated with 1M HCl to activate latent TGF-β, followed by neutralization with 1N NaOH. Following another wash, samples were added to the plate and incubated. Following another wash, biotinylated detection antibody was added to the plate. After another wash, plate was treated with Avidin-HRP, and incubated. After a final wash, plate was treated with 3,3’,5,5’-tetramethylbenzidine substrate (TMB, Moss, Pasadena, MD). After 15 minutes stop solution was added and the plate was read using a Synergy HT Multi-Mode Microplate Reader (Biotek, Winooski, VT) at an absorbance of 450 nm. Cytokine levels were determined by comparison with standard curves generated from recombinant TGF-β after log/log quadratic linear regression analysis using Gen5^TM^ Data Analysis Software (Biotek).

### Determination of *M*. *pulmonis* specific antibody levels

To prepare Ag for ELISA, *M*. *pulmonis* was cultured at 37°C in mycoplasma broth medium for 3 days and harvested. *M*. *pulmonis* broth was adjusted to 5 mg/ml protein concentration. Lysis buffer was added (4.2 g NaHCO_3_/L and 5.3 g Na_2_CO_3_/L, pH 10.0), warmed to 37°C, and added to the *M*. *pulmonis* stock. This was incubated at 37°C for 15 minutes. Then 2.2 g of boric acid was added for every 100 mls lysis buffer, and solution was frozen at -80°C. Protein concentration was determined by Bradford assay.

Falcon 96-well flexible assay plates (BD Biosciences) were coated with optimal concentrations of *M*. *pulmonis* Ag (100 μl at 10 μg/ml) in PBS. After overnight incubation at 4°C, plates were washed three times with PBS-0.05% Tween 20 and blocked overnight at 4°C with 1% milk in PBS. Plates were again washed three times with PBS-0.05% Tween 20. Serum samples were initially diluted 1:400 for IgG and IgM, and 1:200 for IgA. These were then serially diluted 1:2 with 1% milk. 100 μl was placed in each well, and plates were incubated overnight at 4°C. Plates were washed five times with PBS-0.05% Tween 20. Secondary antibody (biotinylated anti-mouse antibody stock reagents of 0.5 mg/ml; Southern Biotechnology Associates, Birmingham, AL) were diluted 1:5000 for IgG and IgM, and 1:2000 for IgA in 1% milk and added to the appropriate wells (100 μl/well). Plates were incubated at room temperature for five hours. Plates were washed three times with PBS-0.05% Tween 20. A 1:2000 dilution of HRP-conjugated streptavidin (neutralite avidin; Southern Biotechnology Associates) in 1% milk was added to each plate (100 μl/well), and plates were incubated for 1 hour at room temperature. Plates were then washed three times with PBS-0.05% Tween 20, and once with PBS. Reaction mixtures were developed at room temperature by addition of 100 μl of 3,3’5,5’-tetramethylbenzidine peroxidase substrate (Moss, Pasadena, CA) in each well. Plates were read using a BioTek Synergy HT plate reader and Gen5 software (BioTek, Winooski, VT) at an absorbance of 630 nm. Endpoint antibody titers were expressed as the reciprocal dilution of the last dilution that gave an OD of 0.1 above the OD of negative controls after 10–15 minute of incubation, as previously described [49, 50]. The negative controls were wells that were treated the same as the other samples, except PBS alone were added instead of serum samples.

### Statistical analysis

Data were evaluated by ANOVA, followed by Tukey’s post-test comparisons. Endpoint titers and mycoplasma CFU results were logarithmically transformed to normalize data, and lesion indices were normalize using arcsine transformation [51]. Data were analyzed by analysis of variance followed by post hoc tests for multigroup comparisons, as needed. Analyses were performed using JMP 10 (SAS Institute, Cary, NC) or Prism software (Graphpad Software, La Jolla, CA). A *p* value ≤ 0.05 was considered statistically significant. Results are expressed as means ± standard errors of the means (SEM).

## Results

### Total numbers of Treg cells increase in the draining lymph nodes during the course of *M*. *pulmonis* infection, and display a classical Treg phenotype

In order to determine the changes in Treg numbers along the lower respiratory tract during mycoplasma disease pathogenesis, mice were intranasally infected with *M*. *pulmonis* and sacrificed at various time points. Cells were isolated from the lungs, lower respiratory lymph nodes (LRN), and spleens. Numbers of CD4^+^CD25^+^FoxP3^+^ Treg cells were determined using flow cytometry. Cells were also stained for the expression of CTLA-4, glucocorticoid-induced TNF-like receptor (GITR), and CD127.

**Fig 1:**
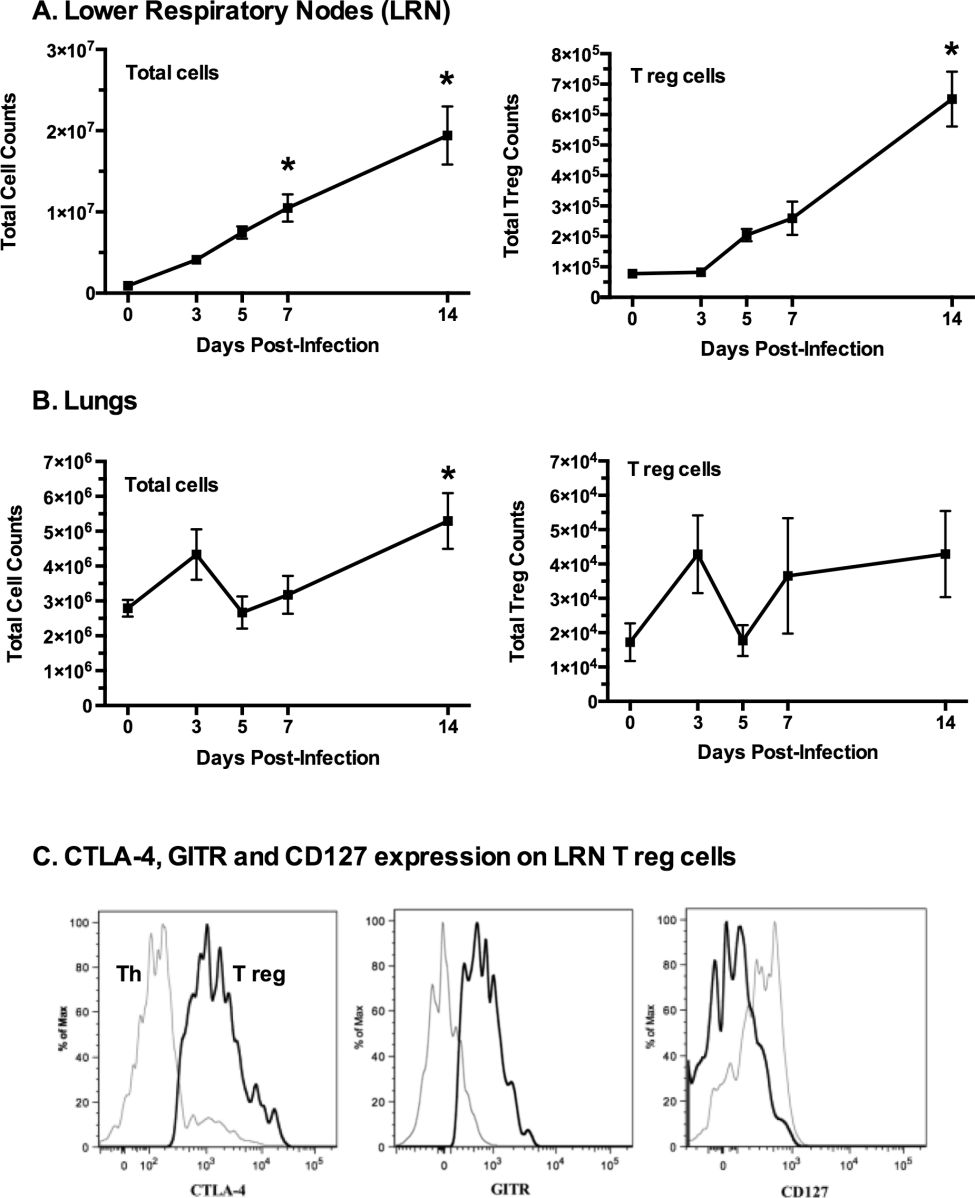
Treg (CD4^+^CD25^+^FoxP3^+^) cells increase after mycoplasma infection. Mice were infected with *M*. *pulmonis* and sacrificed on days 0, 3, 5, 7, and 14 post-infection. Lymphocytes were isolated from (**A**) lower respiratory nodes (LRN) and (**B**) lungs. Treg cells were identified as CD4^+^CD25^+^FoxP3^+^ cells. An asterisk (*) indicates a significant difference (p ≤ 0.05) day 14 cell numbers versus day 0 cell numbers. Error bars represent the mean +/- SE (n = 9). (**C**) The majority of Treg cells in the LRNs were found to express high levels of CTLA-4 and GITR as compared to CD4^+^ effector T cells. In addition, Treg cells displayed little to no CD127 as compared to CD4^+^ cells. The black lines represent Tregs (CD4^+^CD25^+^FoxP3^+^) while gray lines represent CD4^+^CD25^-^FoxP3^-^ Th cells. The histograms are from a representative LRN sample. Experiment was performed three times (n = 9).

The absolute number of CD4^+^CD25^+^FoxP3^+^ T cells in the LRN began to increase by day 5 after infection, reaching almost a three-fold increase by day 7 compared to day 0. This increase continued through day 14 post-infection resulting in more than an eight-fold increase in total CD4^+^CD25^+^FoxP3^+^ T cells (Fig 1). This corresponded with an overall increase in total cells in the LRNs through day 14. Total cell numbers in the lungs also increased significantly between days 5 and 14 post-infection. Treg cells were found to be present in the lungs during infection, but their numbers did not increase significantly. Thus, the numbers of Treg cells increased primarily in the draining lymph nodes, supporting the idea that these cells play a role in the immune response to mycoplasma infection, and that their primary site of action may be the LRN, rather than the lungs.

The majority of CD4^+^CD25^+^FoxP3^+^ T cells in the LRNs were found to express high levels of CTLA-4 and GITR as compared to CD4^+^ effector T cells (Fig 1). The expression levels of these markers on the Treg cells did not significantly change at any time during the infection. Thus, the dominant phenotype of Treg cells in both the lungs and the LRNs during *M*. *pulmonis* infection is CD4^+^CD25^+^FoxP3^+^CTLA-4^+^GITR^+^CD127^lo^, which is the phenotype of classical Treg cells [52–59].

### CD25^+^ cell depletion increases mycoplasma lung disease severity

Mice were depleted of CD25^+^ cells by treatment with anti-CD25 Ab, an approach commonly used to examine Treg cell activity *in vivo* [60, 61], and infected with *M*. *pulmonis*. Control (undepleted) mice were given PBS. Body weights of individual mice were monitored every other day, and mice were sacrificed at 14 days post-infection. Lungs were visually assessed for the presence of gross lesions, and processed for mycoplasma CFU and histopathology. CD25-mediated depletion did not significantly affect the percentage of non-Treg cells (Table 1). One limitation to these studies however was that the day-to-day variations (e.g. 7 days vs 14 days) were sufficient, as well as the number of mice, where small changes due to anti-CD25 antibody treatment would not be recognized. At 14 days, there were about 20% and 50% of CD4^+^CD25^+^FoxP3^+^ cells in LRN and lungs respectively, than those found in control mice, indicating that reduction of cells would remain throughout the course of our studies with infected mice. No significant differences in weight loss or gross lung lesions were observed between PBS control mice and isotype control mice (p > 0.05).

**Table 1:** CD25 antibody-mediated depletion did not significantly affect non-Treg cells.

		Percentage of Live Cells (SD)			
		Control mice		Treg depleted mice	
Cell source	Cell population	Day 7^a^	Day 14	Day 7	Day 14
**LRN**	B220 (B cells)^b^	5.43 (2.4)^c^	9.39 (3.4)	7.84 (2.1)	11.82 (3.3)
	CD11c (DC)	7.50 (4.0)	9.21 (2.7)	7.49 (3.9)	10.19 (2.3)
	F4/80 (Mac)	4.92 (1.0)	2.81 (1.4)	3.67 (0.5)	2.62 (1.5)
	DX5 (NK)	4.88 (2.5)	4.73 (0.8)	3.59 (1.5)	3.96 (0.4)
	CD8 (CTL)	23.97 (22.1)	24.51 (17.5)	15.39 (14.2)	17.37 (11.0)
	CD4 (Th)	46.17 (5.2)	55.83 (9.1)	44.82 (6.3)	49.28 (18.3)
	CD4^+^CD25^+^FoxP3^+^ (Treg)	**----**	4.78 (0.79)	**----**	1.09 (0.10)*
**LUNG**	B220 (B cells)	6.76 (2.6)	5.69 (1.2)	6.40 (2.6)	4.36 (1.0)
	CD11c (DC)	**----**^**d**^	8.66 (0.1)	**----**	7.43 (0.6)
	F4/80 (Mac)	**----**	**----**	**----**	**----**
	DX5 (NK)	**----**	**----**	**----**	**----**
	CD8 (CTL)	1.19 (0.4)	1.52 (0.2)	1.56 (0.6)	1.49 (0.4)
	CD4 (Th)	30.83 (7.3)	42.53 (4.7)	30.78 (8.9)	38.34 (4.8)
	CD4^+^CD25^+^FoxP3^+^ (Treg)	**----**	1.65 (0.12)	**----**	0.91 (0.13)*

^a^Days after infection with *M*. *pulmonis*

^b^Abbreviations: DC- Dendritic Cells; Mac—Macrophages; NK—Natural Killer cells; CTL—Cytotoxic T Lymphocytes; Th—T Helper Cells

^c^Data are expressed as the percentage of live cells as determined by flow cytometry (n = 3).

^d^Data were not collected.

*Significantly different from control mice *P* ≤ 0.05

**Fig 2:**
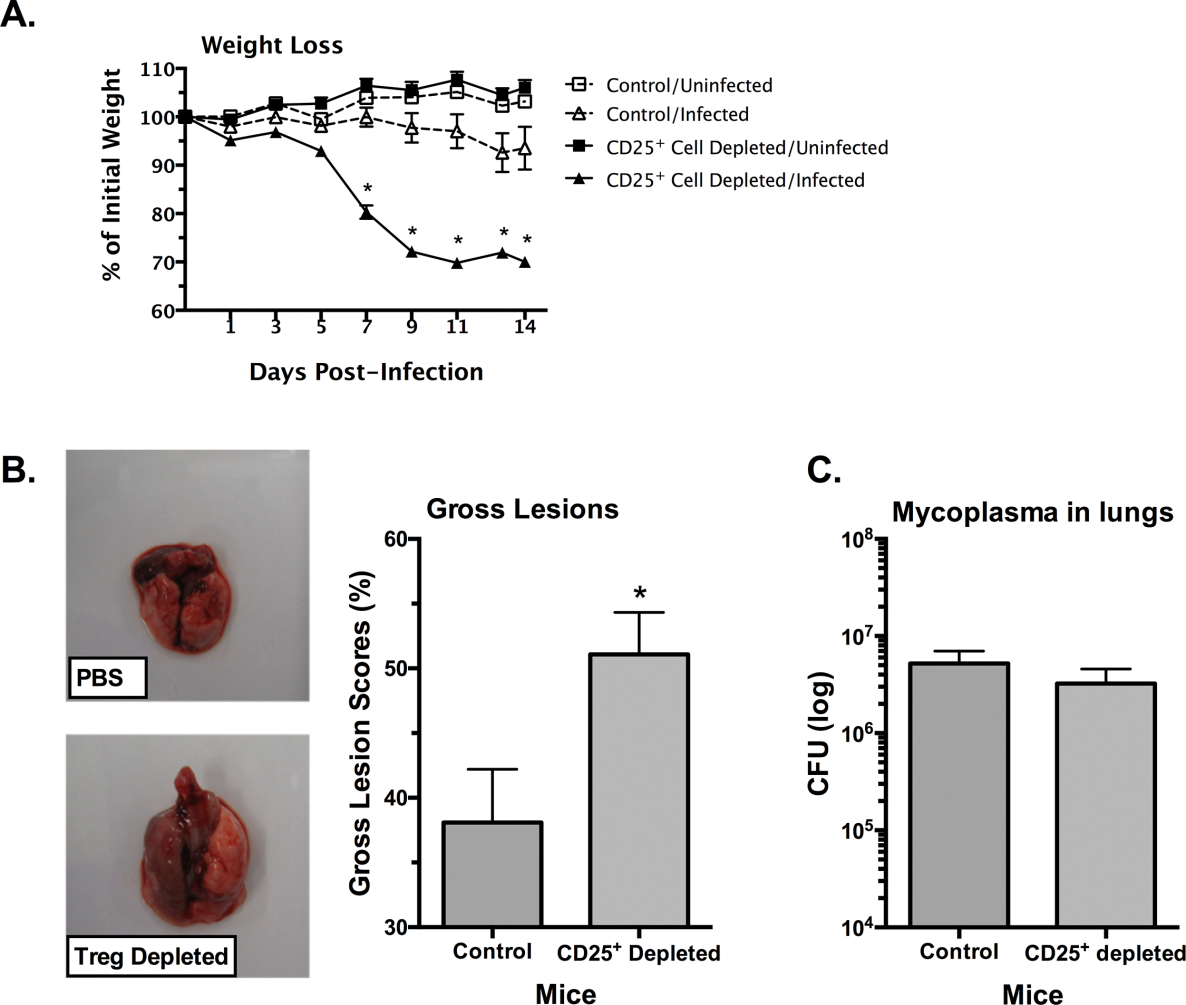
CD25^+^ cell-depleted mice develop more severe disease after mycoplasma infection. Mice were given anti-CD25 depleting antibody (CD25^+^ Cell-Depleted) or PBS (Control) one day prior to infection with *M*. *pulmonis*, followed by antibody or PBS at day 6 post-infection. Uninfected mice were included. (**A**) Weights were monitored every other day. Data are expressed as the percentage of initial weight measured at day 0. Error bars represent SE (n = 8). (**B**) Lungs were visually assessed for the presence of gross lesions on day 14 after infection (n = 8). Representative lungs are shown. The lungs of the PBS-treated mouse were small and display few gross lesions. The lungs of the CD25^+^ cell-depleted mice were larger, and the left lobe appears fully necrotic. An asterisk (*) represents a significant (*P* ≤ 0.05) difference. Vertical bars and error bars represent means +/- SE. (C) The numbers of mycoplasmas in lungs were determined on day 14 after infection (n = 18). There were no differences in the numbers of mycoplasma recovered from the lungs of CD25^+^ cell-depleted mice versus PBS-treated mice. Vertical bars and error bars represent means +/- SE.

**Fig 3:**
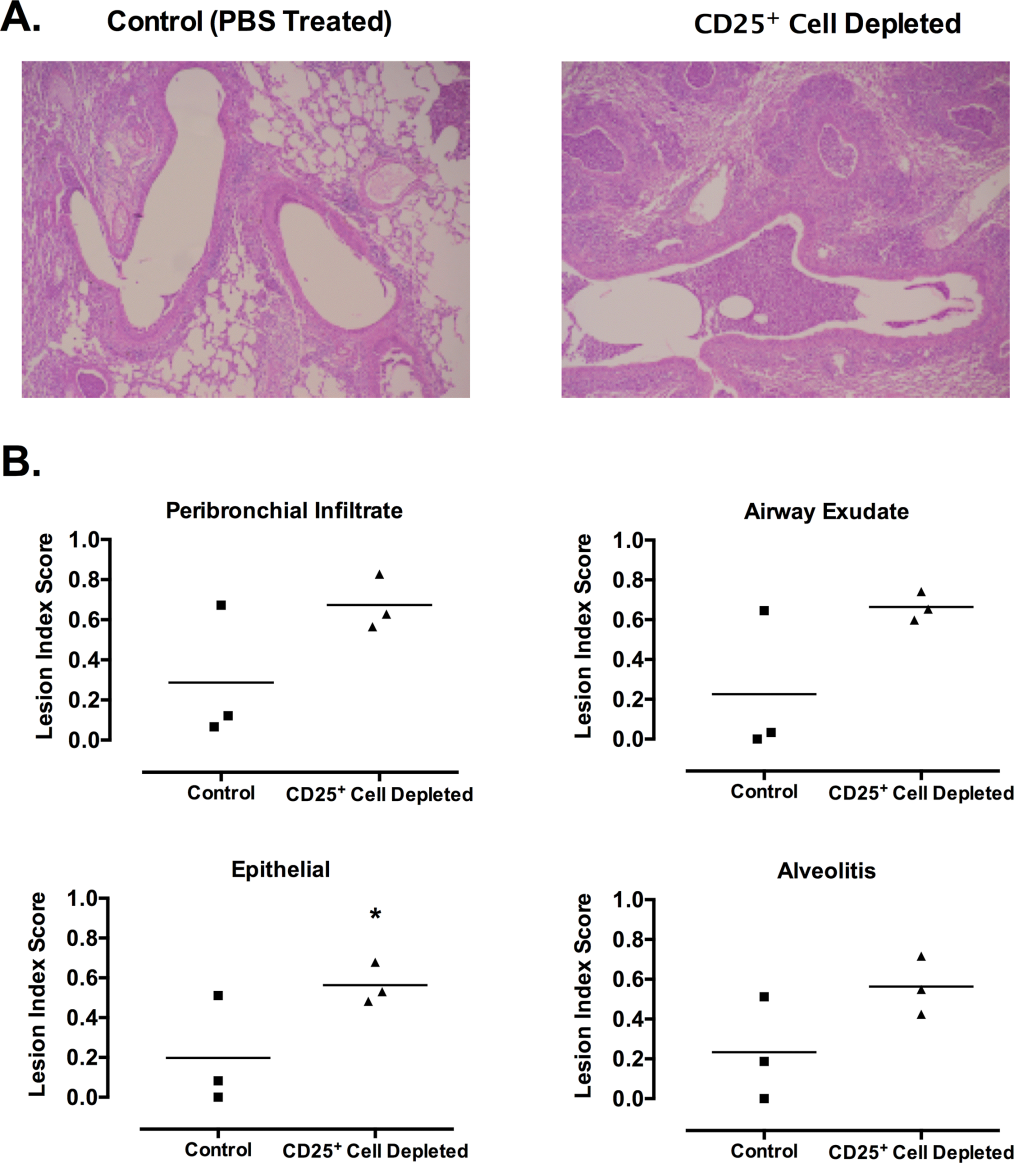
CD25^+^ cell-depleted mice develop more severe histopathology due to mycoplasma infection. Mice were given anti-CD25 depleting antibody (CD25^+^ Cell Depleted) or PBS (Control) one day prior to infection with *M*. *pulmonis*, followed by antibody or PBS at day 6 post-infection. At 14 days post-infection, lungs were fixed for histology. (**A**) Representative sections from lungs of control (PBS treated) and Treg depleted infected mice are shown. (**B**) Lesion index scores for each of the characteristic lesions were determined, e.g. peribronchial and perivascular lymphoid hyperplasia or infiltration (peribronchial infiltrate); neutrophilic exudate in airway lumina (airway exudate); hyperplasia of airway mucosal epithelium (epithelial); and mixed neutrophilic and histiocytic exudate in alveoli (alveolitis). Individual data points and horizontal lines representing the means are shown (n = 3). An asterisk (*) represents a significant (*P* ≤ 0.05) difference. Overall, there was a significant (*P* ≤ 0.05) increase in the overall severity of lung lesions of mycoplasma disease when CD25^+^ T cells were depleted [PBS 0.24 +/-0.06; CD25^+^ Cell Depleted 0.62 +/- 0.06 (Mean +/- SE)].

CD25^+^ cell depletion caused significantly more severe disease in infected mice. Mice that were CD25^+^ cell-depleted lost significantly more weight over the course of the infection as compared to mice that received no depleting antibody (Fig 2). Mice that were CD25^+^ cell-depleted and subsequently infected lost greater than 30% of their initial body weight, while infected mice that received no CD25^+^ cell-depleting antibody lost less than 7% of their body weight. There was also an increase in pulmonary disease in mycoplasma-infected mice when treated with anti-CD25 antibody. The lungs of CD25^+^ cell-depleted mice had increased severity of gross lesions as compared to mice that were not CD25-depleted. Furthermore, histopathologic examination showed that CD25^+^ cell-depleted mice had overall more severe lesions (*P* ≤ 0.05) [PBS 0.24 +/-0.06; CD25^+^ Cell Depleted 0.62 +/- 0.06 (Mean +/- SE)] relative to infected, non-depleted mice. There were higher lesion indices for each of the inflammatory lesion types examined, e.g. peribronchial infiltration, neutrophilic airway exudate, epithelial hyperplasia, and alveolitis, in CD25^+^ cell depleted mice, but these differences were not significant, except for epithelial hyperplasia, most likely due to low number of replicates within these experiments (Fig 3, n = 3). Overall, there was an increase in all the characteristic lesions of mycoplasma disease in the absence of CD25^+^ cell cells, suggesting a role of Treg cells in dampening formation of inflammatory lesions in the lungs of infected mice.

Interestingly, there were no significant differences in the numbers of mycoplasmas recovered from the lungs of CD25^+^ cell-depleted and control mice after mycoplasma infection (Fig 2C). Thus, the increase in disease severity seen in CD25^+^ cell-depleted mice did not correspond with a change in the bacterial burden in the lungs. This suggests that the increased disease was not due to a change in the levels of bacteria, but rather to increased inflammatory reactions.

### CD25^+^ cell depletion coupled with *M*. *pulmonis* infection causes increased lung cell infiltration, and increased serum antibody responses

The previous studies demonstrated that mycoplasma disease is more severe in the absence of CD25^+^ regulatory T cells, correlating with increased infiltration of lymphocytes along the airways. In addition, the increased severity of clinical signs corresponded with the time point when adaptive immunity has previously been shown to become apparent. To examine whether there was a preferential change in mononuclear cell populations or antibody responses against mycoplasma in the absence of CD25^+^ cells, mice were treated with anti-CD25 Ab and infected with *M*. *pulmonis*. Mice were sacrificed at days 7 and 14 post-infection, and cells were isolated from spleens, LRNs, and lungs. In addition, blood was collected, and serum was used in ELISAs to measure levels of mycoplasma-specific IgG, IgM, and IgA.

**Fig 4:**
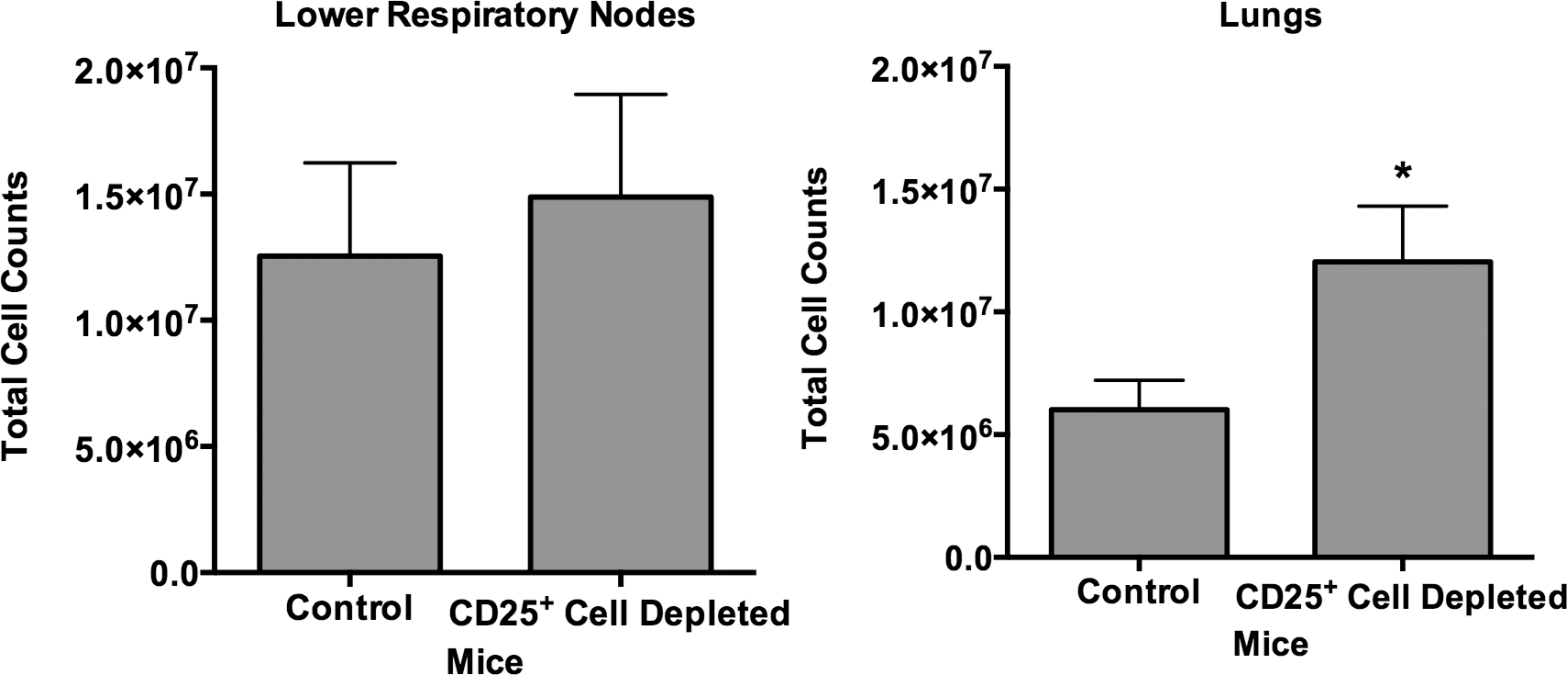
CD25^+^ cell-depletion results in increased lung cell infiltration. Mice were given anti-CD25 depleting antibody (CD25+ Cell Depleted) or PBS (Control) one day prior to infection with *M*. *pulmonis*, followed by antibody or PBS at day 6 post-infection. At 14 days post-infection, total cell counts in the LRN and lungs were determined. Vertical bars and error bars represent means +/- SE (n = 8). An asterisk (*) represents a significant (*P* ≤ 0.05) difference between the numbers of cells recovered between the two groups.

CD25^+^ cell-depletion led to an increase in the total number of mononuclear cells in the lungs by day 14 post-infection as compared to mice that received no depleting antibody (Fig 4). In contrast, total cells in the LRN of infected CD25^+^ cell-depleted mice were not significantly higher compared to infected non-depleted mice. The increases in cell counts did not appear to be due to the expansion of any specific cell population, as no significant changes in the percentages of B or T cell populations recovered from the lungs or LRN (Table 1). Thus, the depletion of CD25^+^ regulatory T cells resulted in the increased infiltration of lymphocyte populations equally.

**Fig 5:**
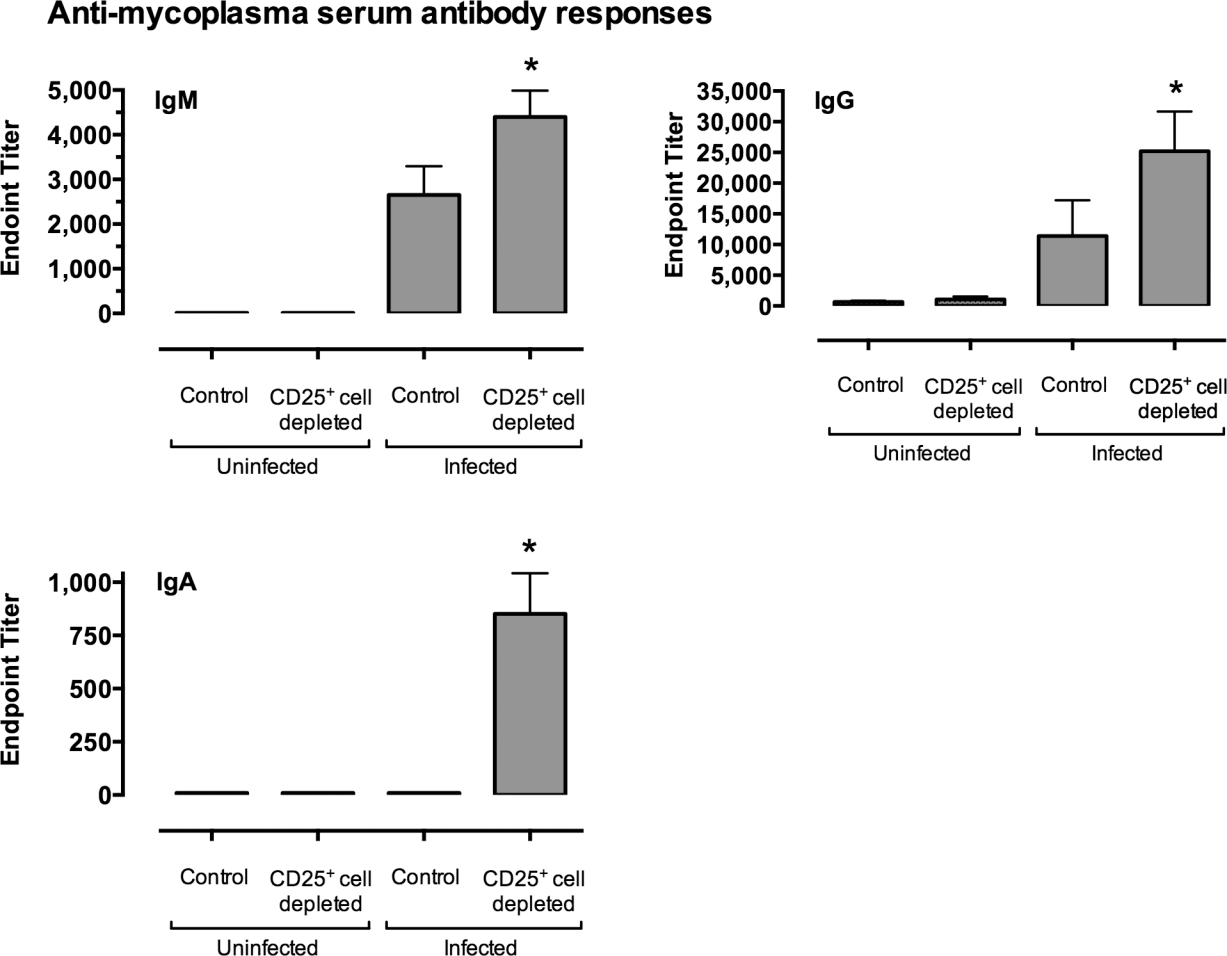
Mycoplasma-specific serum antibody responses were higher in infected CD25^+^ cell-depleted mice as compared to infected control mice. Mice were given anti-CD25 depleting antibody (CD25^+^ Cell Depleted) or PBS (Control) one day prior to infection with *M*. *pulmonis*, followed by antibody or PBS at day 6 post-infection. Uninfected mice were included for comparison. At 14 days post-infection, *M*. *pulmonis* specific titers for IgM, IgG and IgA were determined. Data were analyzed after log transformation. An asterisk (*) represents a significant (*P* ≤ 0.05) difference antibody levels in CD25^+^ cell-depleted mice versus the other groups. Vertical bars and error bars represent means +/- SE (n = 16).

Serum levels of mycoplasma-specific IgG, IgM, and IgA were all significantly higher in infected CD25^+^ cell-depleted mice as compared to infected non-depleted mice (Fig 5). These data show that CD25^+^ cell-depletion prior to infection leads to an increase in the overall magnitude of the immune and inflammatory response, as indicated by higher lung cell counts and higher levels of mycoplasma-specific serum antibodies, and therefore, these cell likely act by dampening harmful inflammatory responses, previously shown to be mediated by Th cells [7].

### CD25^+^ cell-depletion skews the immune response towards a Th2 phenotype

Treg cells alter the activation of Th cells, either by dampening all T cell activation or shifting the Th1/Th2 balance [62–67]. Previous data from our lab indicate that Th2 responses contribute to immunopathology [10]. To examine whether regulatory T cells altered the activation of Th cells, mice were depleted of CD25^+^ cells and infected with *M*. *pulmonis*. Mice were sacrificed at 7 days after infection, and CD4^+^ cells from LRNs and lungs were stained for intracellular IFN-γ and IL-13.

**Fig 6:**
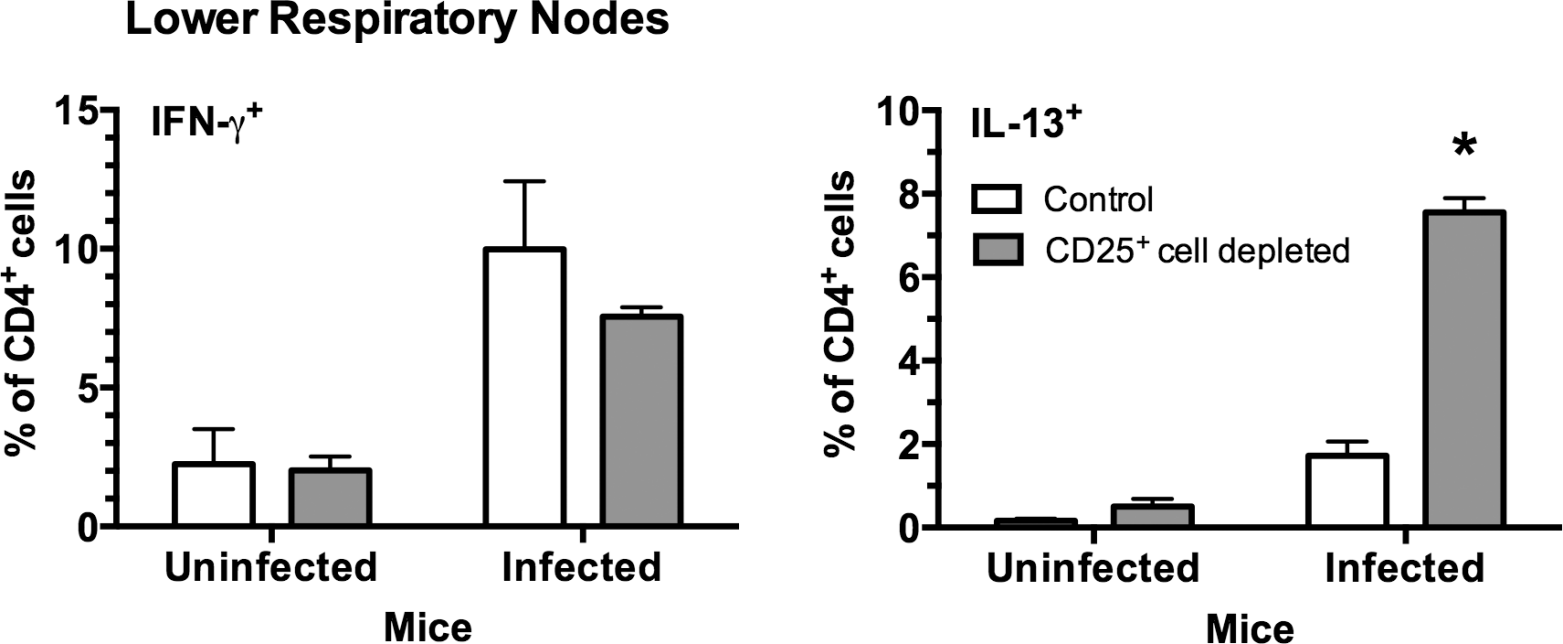
CD25^+^ cell-depletion resulted in higher levels of IL-13^+^ CD4^+^ T cells in the LRNs of infected mice. Mice were given anti-CD25 depleting antibody (CD25^+^ cell-depleted) or PBS (Control) one day prior to infection with *M*. *pulmonis*, followed by antibody or PBS at day 6 post-infection. As controls, uninfected mice were similarly treated. Seven days after infection, the percentage of IFN-γ and IL-13 expressing CD4^+^ T cells in the LRNs of *M*. *pulmonis* infected mice were examined using flow cytometry. An asterisk represents a significant (*P* ≤ 0.05) difference IL-13 versus IFN-γ. Vertical bars and error bars represent means +/- SE (n = 5).

Surprisingly, CD25^+^ cell-depletion prior to infection caused a shift in the immune response towards Th2, as shown by an increase in the levels of IL-13^+^ CD4^+^ T cells and a slight decrease in IFN-γ^+^ CD4^+^ T cells in the LRNs (Fig 6). These data suggest Treg or similar cells dampen activation of Th2 responses generated in the lymph nodes, which may subsequently influence disease severity.

### Antigen-specific CD4^+^CD25^+^ T cells stimulate the *in vitro* production of IFN-γ and IL-17 by CD4^+^ T cells, but not IL-4, IL-10 or TGF-ß

To further examine the possibility that Treg cells could modulate mycoplasma-specific lymphocyte responses, mice were infected with *M*. *pulmonis*. Cells from LRN were harvested at 8 days post-infection. Cells were sorted into CD4^+^CD25^+^ T cells, and CD4^+^CD25^-^ Th cells. Th cells and antigen-presenting cells (CD3-depleted naïve splenocytes) were cultured *in vitro* with or without CD4^+^CD25^+^ T cells. The cells were stimulated with mycoplasma Ag. Four days later, supernatants were assayed for IL-4, IL-10, IL-13, IL-17, TGF-β, and IFN-γ.

**Fig 7:**
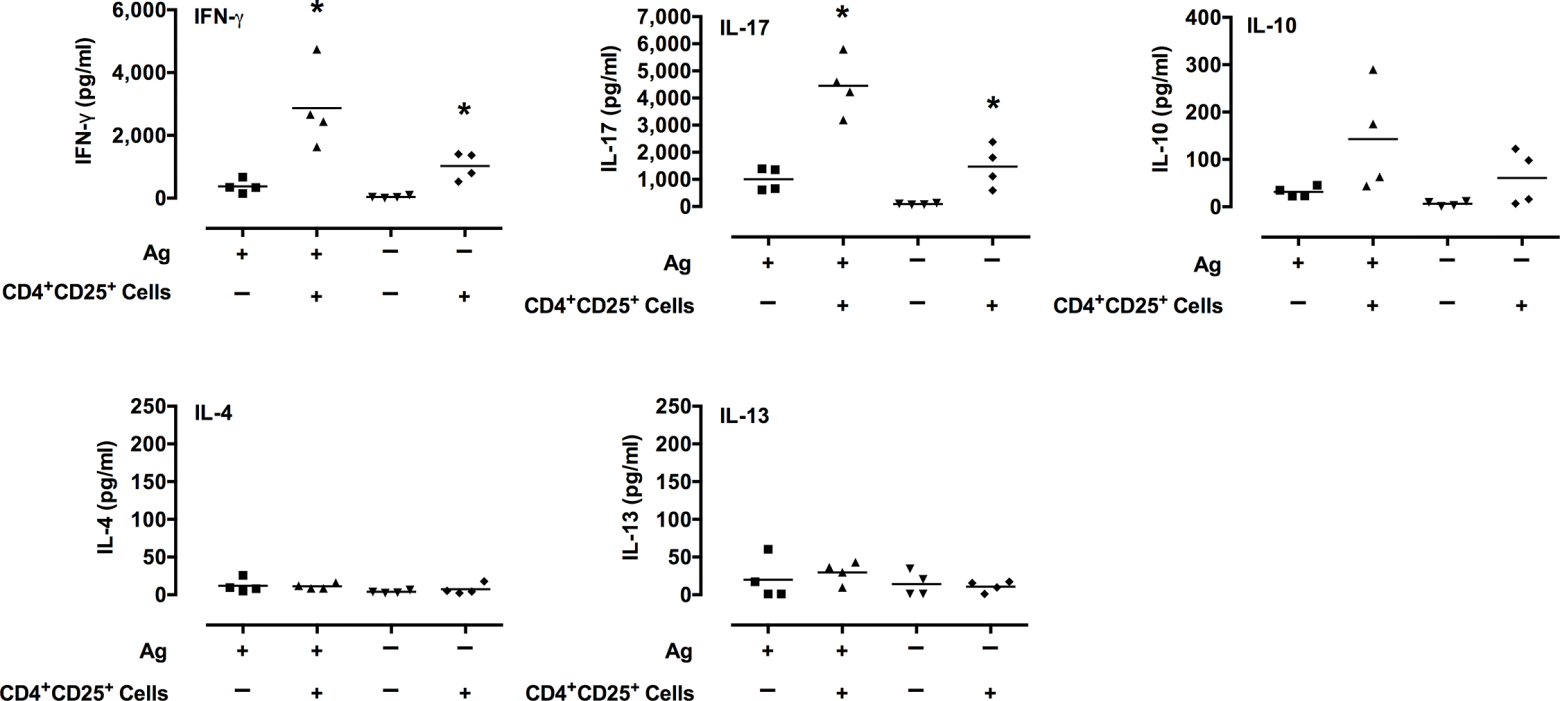
CD4^+^CD25^+^ T cells stimulate the *in vitro* production of IFN-γ and IL-17 by CD4^+^ T cells. CD4^+^CD25^+^ T cells and CD4^+^CD25^-^ Th cells from LRN of infected mice were isolated. Th cells and Ag-presenting cells (CD3-depleted naïve splenocytes) were cultured *in vitro* with or without CD4^+^CD25^+^ T cells. The cells were cultured in the presence or absence of mycoplasma Ag as indicated. Four days later, supernatants were assayed for IL-4, IL-10, IL-13, IL-17, TGF-β, and IFN-γ. TGF-β, IL-4 and IL-13 levels were low and did not increase in cultures stimulated with mycoplasma Ag. An asterisk (*) represents a significant (*P* ≤ 0.05) difference (Ag + CD4^+^CD25^+^ T cells versus Ag only). Individual data points and horizontal lines representing the means are shown (n = 4). A graph for TGF-β levels was not included, as all samples were below the level of detection (< 62.5 pg/ml) using an ELISA.

Surprisingly, the addition of CD4^+^CD25^+^ T cells led to significant increases in IL-17 and IFN-γ production in response to mycoplasma Ag (Fig 7). There appears to also have been an increase in IL-10, but these responses were variable and not statistically significant. TGF-β, IL-4, and IL-13 levels were low and did not increase in cultures stimulated with mycoplasma Ag at 4 days in culture. It is possible that low levels of these cytokines appear at earlier time points, but previous studies do not suggest this as a possibility. In addition, the presence of CD4^+^CD25^+^ T cells led to a significant increase in IL-17 even in the absence of any stimulation. In preliminary studies, cultures that did not include Th cells, there were lower levels of IL-17 and little to no IFN-γ or IL-10 in culture supernatants after stimulation with mycoplasma Ag (S1 Fig), indicating that the CD4^+^CD25^+^ T cells were not the major source of these responses. These data suggest that CD4^+^CD25^+^ regulatory T cells promote the secretion of IFN-γ and IL-17 by Th cells, but further studies are needed. Furthermore, the increases in these cytokines in response to mycoplasma Ag indicate that a component of this response is antigen-specific.

### Depletion of CD25^+^ cells decreased IFN-γ^+^ and IL-17^+^ Th cells in the LRN of infected mice

The previous studies demonstrated that Treg cells could enhance mycoplasma-specific IFN-γ and IL-17 responses by Th cells *in vitro*. To determine whether CD25^+^ regulatory T cells have a similar effect *in vivo*, mice were treated with anti-CD25 depleting antibody or PBS control 1 day prior to infection with *M*. *pulmonis* and followed by antibody or PBS at day 6 post-infection. Mice were sacrificed at days 7 post-infection, and cells were harvested from LRN and lungs. Cells were stained for CD4, CD25, FoxP3, IFN-γ and IL-17.

**Fig 8:**
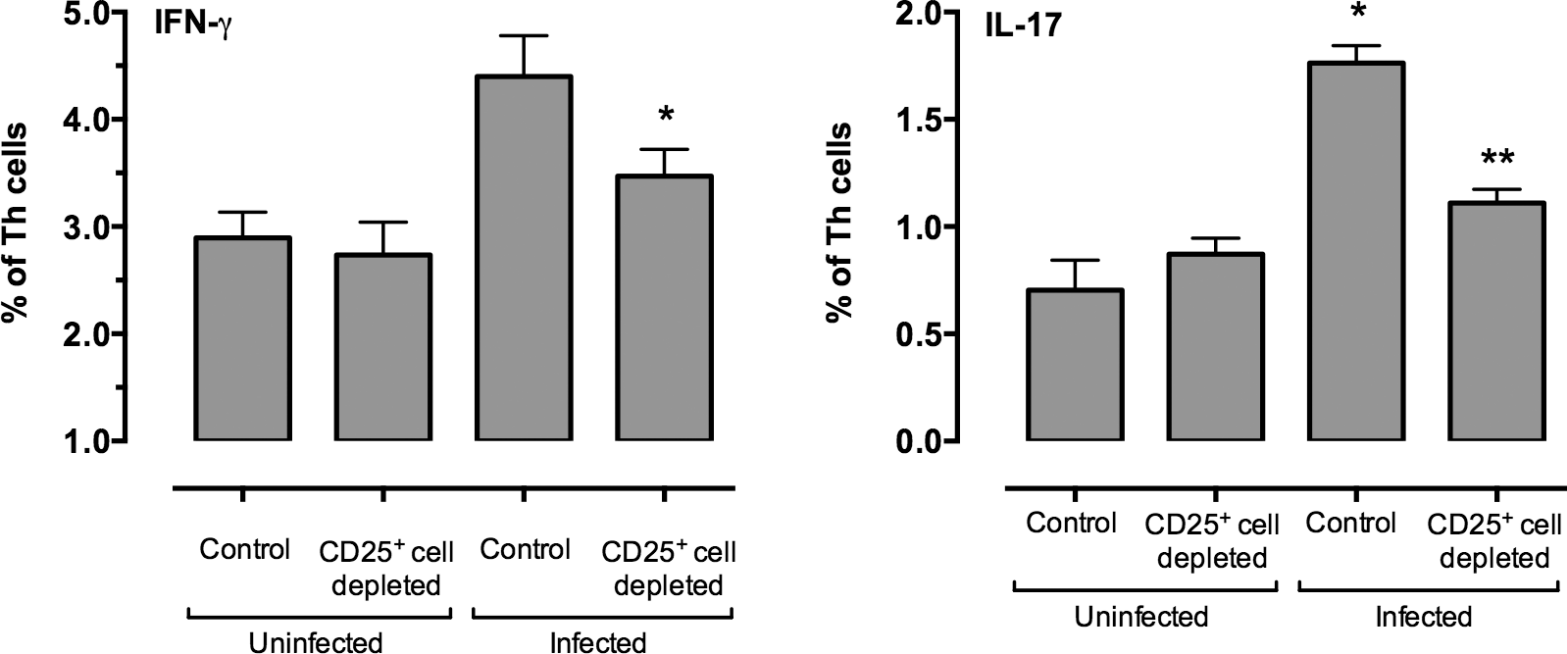
Depletion of CD25^+^ cells decreased the percentage of IL-17 and IFN-γ CD4^+^ Th cells in the LRN of infected mice. Mice were given anti-CD25 depleting antibody (CD25^+^ cell depleted) or PBS (Control) one day prior to infection with *M*. *pulmonis*, followed by antibody or PBS at day 6 post-infection. Mice were sacrificed at day 7 after infection, and cells harvested from LRNs. Data are expressed as a percentage of CD4^+^CD25^-^FoxP3^-^ lymphocytes (Th cells). An asterisk (*) represents a significant (*P* ≤ 0.05) difference between the percentages of cells found in lymph nodes of control and Treg depleted mice. A double asterisk (**) represents a significant (*P* ≤ 0.05) difference infected mice that were depleted of CD25^+^ cells and control (PBS-treated) mice. Vertical bars and error bars represent means +/- SE (n = 6).

CD25^+^ cell-depletion resulted in a decrease in the percentage of IFN-γ^+^ and IL-17^+^ Th cells in the LRN at day 7 post-infection (Fig 8). There was no effect on these cells in uninfected (control) mice. Consistent with the results from the *in vitro* studies, these data support the idea that CD25^+^ regulatory T cells, enhance Th1 and Th17 type responses against mycoplasma *in vivo*.

### IL-17^+^ and IFN-γ^+^ populations of CD4^+^CD25^+^FoxP3^+^ (Treg) T cells expand in response to mycoplasma infection

To confirm that IL-17^+^ and/or IFN-γ producing Treg cells developed in response to mycoplasma infection, mice were infected with *M*. *pulmonis*, and cells were isolated from lungs and LRN after infection. The cells were immunofluorescently stained for the expression of CD4 and CD25, and intracellular FoxP3, IL-17, and IFN-γ.

**Fig 9:**
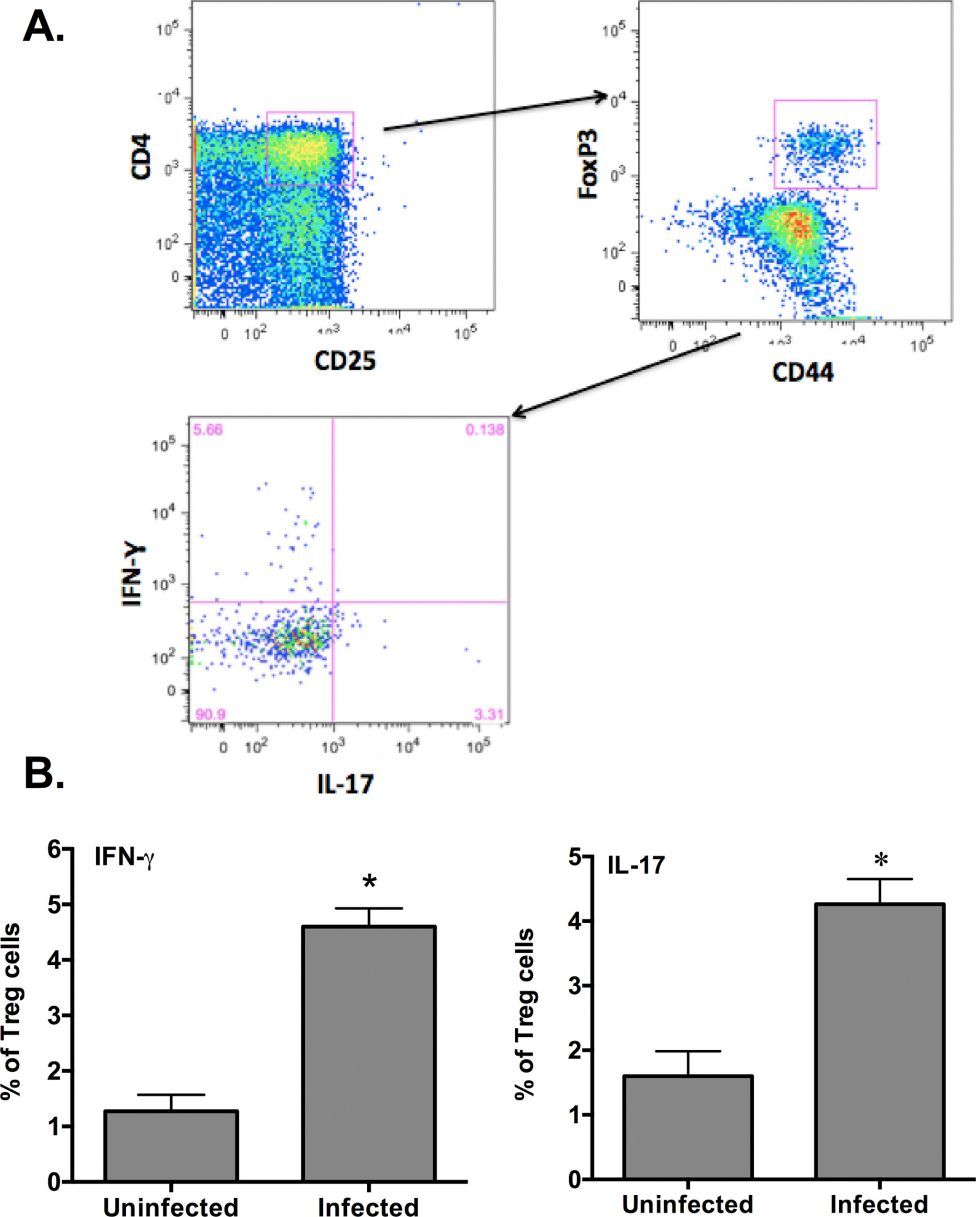
IL-17^+^ and IFN-γ^+^ populations of Treg (CD4^+^CD25^+^FoxP3^+^) cells expand in LRN after mycoplasma infection. Mice were infected with *M*. *pulmonis*, and 7 days later, cells were isolated from LRN after infection. The cells were immunofluorescently stained for the expression of CD4 and CD25, and intracellular FoxP3, IL-17 and IFN-γ. (**A**) CD4^+^CD25^+^FoxP3^+^ CD44^+^ (Treg) cells were identified and gated for determination of IL-17 and IFN-γ expressing cells. Double positive (IFN-γ^+^IL-17^+^) Treg cells were not found. (**B**) The percentage of IFN-γ^+^ Treg cells and IL-17^+^ Treg cells increased in the LRN of mycoplasma infected mice. No changes in these cell populations were found in lungs (*P* > 0.05). An asterisk (*) represents a significant (*P* ≤ 0.05) difference between infected versus uninfected mice. Vertical bars and error bars represent means +/- SE (n = 6).

Populations of IFN-γ^+^ CD4^+^CD25^+^FoxP3^+^ (Treg) T cells and IL-17^+^ Treg cells were found in the LRN of mycoplasma infected mice (Fig 9). The percentage of both IFN-γ-expressing Treg cells and IL-17-expressing Treg cells significantly increased in the LRNs of infected mice relative to uninfected mice at day 7 post-infection. There were no double positive (IFN-γ^+^IL-17^+^) Treg cells observed in either organ, nor were IL-10^+^ Treg cells. These data demonstrate that IFN-γ-expressing and IL-17-expressing CD4^+^CD25^+^FoxP3^+^ (Treg) T cells subpopulations are present and expand in LRN after mycoplasma infection.

## Discussion

Mycoplasma respiratory disease is immunopathologic [46,47]. It is clear that elements of the adaptive immune response contribute to the pathology, while some responses are protective against *M*. *pulmonis* infections. Pulmonary T cell activation, and the mechanisms that regulate these responses, is clearly instrumental in the pathogenesis of mycoplasma respiratory disease of the lower respiratory tract [14,48]. Although other cell populations can modulate mycoplasma disease [7, 11, 12], the role of regulatory T cell populations, such as Treg cells, in mycoplasma respiratory diseases has not yet been examined. There has been limited work examining the role of Treg or other regulatory T cells in the pathogenesis of bacterial lung diseases. It is clear that modulation of regulatory T cell activity in some cases benefits the host but in other cases benefits the pathogen [33–35]. Since the lung is a critical organ and a common site for exposure to infection, regulatory T cell activity can benefit the host by dampening inflammatory responses to infections in order to minimize damage to the lung tissue. These regulatory T cells may also promote persistence of infections through the suppression of innate immune cell activity that would otherwise help control or clear infection, which would benefit the pathogen. The role of Treg and other regulatory T cells on immunity and progression of infection depends largely on the specific microorganism and the immune mechanisms involved. In the current study, the role of CD25^+^ regulatory T cells, such as Treg cells, in mycoplasma respiratory diseases was examined in BALB/c mice. Given the persistence of mycoplasma infections and the development of chronic inflammatory lesions, it was hypothesized that Treg cells or other CD25^+^ regulatory T cells control the severity of the inflammatory lesions through production of IL-10 or TGF-ß, but in doing so, these cells could inadvertently promote persistence of infection, as found in other diseases.

Regulatory T cells, most likely a population of Treg cells, clearly control the severity of disease in mycoplasma respiratory infection. Treg cells (CD4^+^CD25^+^FoxP3^+^) preferentially expanded in the host after mycoplasma infection, suggesting that they do play a role in the disease. Further characterization of the Treg cells in mycoplasma-infected mice showed that they express high levels of both CTLA-4 and glucocorticoid-induced TNF-like receptor (GITR), and low levels of CD127, which is the expression pattern corresponding to traditional Treg cells [52–59]. Importantly, depletion of regulatory T cells using an anti-CD25 cell-depleting antibody prior to infection with *M*. *pulmonis* resulted in significant increases in disease severity, as measured by weight loss, gross lung lesions, and histological lung lesions. This effect corresponded with a depletion of CD4^+^CD25^+^FoxP3^+^ cells, a phenotype consistent with Treg cells. Increased disease in CD25^+^ cell-depleted mice also paralleled increases in both lung cell infiltration and mycoplasma-specific serum antibody titers. However, it is possible that anti-CD25 antibody treatment may also deplete effector T cell populations [68]; however, there was an increased inflammatory response and higher antibody response, indicating that immune responses were enhanced after antibody treatment as a result of depleting a regulatory cell population. As susceptibility/resistance to mycoplasma disease can differ between mouse strains [69], regulatory T cells may have varying impact between the different strains of mice. Presumably, regulatory T cells will have similar effects in mice that have similar susceptibility as BALB/c mice, whereas these cells will have little or different roles more resistant strains of mice. For example, C57BL/6 mice have lower numbers of Treg cells than BALB/c mice [70] and are resistant to mycoplasma disease [71]. As resistance to disease in C57Bl/6 mice is dependent upon alveolar macrophage activity and independent of T cells [5, 72, 73], Treg cells most likely have little impact on lung disease progression in this mouse strain. Overall, our results demonstrate that a regulatory CD4^+^CD25^+^FoxP3^+^ T cell population(s) plays an important role in dampening inflammation and immunopathology in mycoplasma respiratory disease in susceptible BALB/c mice, and most likely the population mediating this effect includes Treg cells.

These regulatory T cells did not contribute to the persistence of mycoplasma infection. There are examples where Treg cells and/or anti-inflammatory cytokines, e.g. IL-10, appear to dampen innate host responses to a level that interferes with the normal clearance of a persistent infection [38–42, 74–77]. In these cases, the depletion of Treg cells or inhibition of the anti-inflammatory cytokines results in a greater inflammatory response, but clearance of the organisms is still achieved. In contrast to these examples, we did not find a change in the numbers of mycoplasma recovered from the lung after depletion of CD25^+^ cells. This is despite the appearance of an increased inflammatory response. Thus, Treg cells or other CD25^+^ regulatory T cells modulate host responses contributing to the development of inflammatory lung lesions, but the dampening of these inflammatory responses do not significantly impact the persistence of the mycoplasma infection.

Treg cells in the LRN, rather than the lungs, most likely have the greatest impact on the development of inflammatory responses along the airways of mycoplasma-infected mice. The idea that Treg cells mediate much of their activity in draining lymph nodes (e.g. LRN) is consistent with previous studies [78–81]. In the present study, there were increases in Treg cell numbers in LRN, but not in the lungs of mice infected with mycoplasma. *In vivo* depletion of CD25^+^ cells led to a decrease in IFN-γ^+^ and IL-17^+^ Th cells, while IL-13^+^ Th cells increased; this shift in Th cell populations was seen in cells in LRN but not in the lungs. The preferential expansion and altered activation of CD4^+^CD25^+^FoxP3^+^ (Treg) T cells in LRN of mycoplasma-infected mice supports the idea that LRN are the major site where Treg cells mediate their activity. Importantly, this alteration of Th cell activity may play a significant role in the pathogenesis of mycoplasma disease [82]. In fact, previous studies showed that Th cells and the balance of Th1/Th2 responses during the pathogenesis of mycoplasma infection impacts disease [10, 11]. Th2 cells appear to increase disease severity, and the current studies indicate that there is a shift of Th cell responses towards Th2 after depletion of CD25^+^ cells. Th1 cells on the other hand foster less severe disease and can participate in the control or resistance to infection. Several studies [83–85] also found that Treg cells preferentially can impact the development, functional activation, and proliferation of Th2 cells. Thus, it appears that Treg or related cells in LRN dampen the severity of mycoplasma immunopathology through their modulation of Th cell subset responses, but further studies are needed.

The mechanisms through which Treg cells in mycoplasma disease modulate Th2 inflammatory responses are currently unclear, but our study suggests that there are different mechanisms involved. Treg cells are often thought to function through the production of anti-inflammatory cytokines, e.g. IL-10 and/or TGF-ß [14–21]. However, the current study found no evidence of the production of IL-10 by CD4^+^CD25^+^ T cells from mycoplasma-infected mice after *in vitro* Ag stimulation or CD4^+^CD25^+^FoxP3^+^ T cells after intracellular cytokine staining. It is possible that these cells could promote IL-10 production by other cells, and therefore, regulatory T cells could contribute to dampening of inflammatory responses indirectly through IL-10 production. Interestingly, CD4^+^CD25^+^FoxP3^+^ T cells included two subpopulations that expressed either IFN-γ or IL-17. The presence of IFN-γ and IL-17 production by Treg cells was previously described in mice [28] and humans [23, 26]. To our knowledge, though, this is the first time that IFN-γ^+^ or IL-17^+^ CD4^+^CD25^+^FoxP3^+^ T cells were found in an animal with disease due to a natural pathogen, and the first observation of IFN-γ^+^ or IL-17^+^ CD4^+^CD25^+^FoxP3^+^ T cells with specificity for a foreign antigen. The significance of these cells remains to be defined. However, *in vitro* cultures containing CD4^+^CD25^+^ T cells and CD25^-^ Th cells from LRNs of infected mice secreted significantly higher levels of IFN-γ and IL-17 when stimulated with mycoplasma Ag as compared to CD25^-^ Th cells alone. These results are consistent with the *in vivo* studies where depletion of CD25^+^ cells resulted in a decrease in IFN-γ and IL-17 responses by Th cells within the LRN. Enhancement of IL-17 responses by Treg cells occurs in a wide variety of infections in mice [86–89], and in some cases, the promotion of IL-17 responses results in less severe disease and reduced infection, while in others the increased Th17 responses enhances disease pathology. While IFN-γ is well established as a Th1-type cytokine, the role of IL-17 is less clear, as it is not traditionally associated with either Th1 or Th2 responses. However, Bai et al. recently demonstrated that IL-17 could promote Th1 responses in *C*. *muridarum* lung infection [90]. Thus, we suggest that regulatory T cells in mycoplasma-infected mice may dampen Th2 responses in part by promoting Th cells to secrete IFN-γ and/or IL-17 [90, 91], resulting in decreased immunopathology by shifting the balance of the Th cell subset response.

In conclusion, CD25^+^ regulatory T cells play an important role in controlling damaging immune responses in mycoplasma respiratory infection but do not contribute to the persistence of mycoplasma infection in BALB/c mice. Although the data strongly support that a CD4^+^CD25^+^FoxP3^+^ Treg cell is responsible, there is a possibility that another CD25^+^ regulatory T cell population is involved. In any case, these regulatory T cells reduce the severity of disease and lymphocyte infiltration into the lung, and these cells also suppress antibody responses against the organism. Suppression of these responses is not associated with the production of anti-inflammatory cytokines IL-10 and TGF-ß. In contrast, the anti-mycoplasma regulatory T cells appear to shift responses away from Th2-mediated immunopathologies and increase the production of IFN-γ and IL-17, altering the mycoplasma T cell responses. The presence of IFN-γ^+^ and IL-17^+^ Treg cells, and a lack of IL-10 or TGF-ß expressing Treg cells, suggest that novel regulatory T cell populations are contributing to the shift away from immunopathologic responses in chronic mycoplasma respiratory disease. It is possible that further understanding of the mechanisms that influence disease severity will result in development of therapies that can prevent or decrease immunopathologic damage in lung diseases, or lead to approaches to minimize adverse reactions due to vaccination.

## Supporting Information

S1 Fig *In vitro* stimulation of CD4^+^CD25^+^ T cells results in *in vitro* production of lower levels of IL-17. In preliminary studies, CD4^+^CD25^+^ T cells from LRN of infected mice were isolated. These cells were co-cultured with Ag-presenting cells (CD3-depleted naïve splenocytes) with or without mycoplasma Ag. Four days later, supernatants were assayed for IL-4, IL-10, IL-13, IL-17, TGF-β, and IFN-γ. Lower levels of IL-17 were produced, as compared to cultures containing CD4^+^CD25^-^ T cells; note scale differences between this figure and those in Fig 7. Little to no IFN-γ, TGF-β, IL-4 or IL-13 were produced in response to mycoplasma Ag. Individual data points and horizontal lines representing the means are shown (n = 2).
(TIFF)

## Abbreviations

Ag: Antigen
LRN: Lower respiratory nodes
Treg: T regulatory cells
